# Progress and challenges of developing volatile metabolites from exhaled breath as a biomarker platform

**DOI:** 10.1007/s11306-024-02142-x

**Published:** 2024-07-08

**Authors:** Hsuan Chou, Lucy Godbeer, Max Allsworth, Billy Boyle, Madeleine L. Ball

**Affiliations:** Owlstone Medical Ltd., Cambridge, UK

**Keywords:** Breath analysis, Volatile organic compounds, Breath biomarkers, Breathomics, Volatilomics

## Abstract

**Background:**

The multitude of metabolites generated by physiological processes in the body can serve as valuable biomarkers for many clinical purposes. They can provide a window into relevant metabolic pathways for health and disease, as well as be candidate therapeutic targets. A subset of these metabolites generated in the human body are volatile, known as volatile organic compounds (VOCs), which can be detected in exhaled breath. These can diffuse from their point of origin throughout the body into the bloodstream and exchange into the air in the lungs. For this reason, breath VOC analysis has become a focus of biomedical research hoping to translate new useful biomarkers by taking advantage of the non-invasive nature of breath sampling, as well as the rapid rate of collection over short periods of time that can occur. Despite the promise of breath analysis as an additional platform for metabolomic analysis, no VOC breath biomarkers have successfully been implemented into a clinical setting as of the time of this review.

**Aim of review:**

This review aims to summarize the progress made to address the major methodological challenges, including standardization, that have historically limited the translation of breath VOC biomarkers into the clinic. We highlight what steps can be taken to improve these issues within new and ongoing breath research to promote the successful development of the VOCs in breath as a robust source of candidate biomarkers. We also highlight key recent papers across select fields, critically reviewing the progress made in the past few years to advance breath research.

**Key scientific concepts of review:**

VOCs are a set of metabolites that can be sampled in exhaled breath to act as advantageous biomarkers in a variety of clinical contexts.

## Introduction

**Fig. 1:**
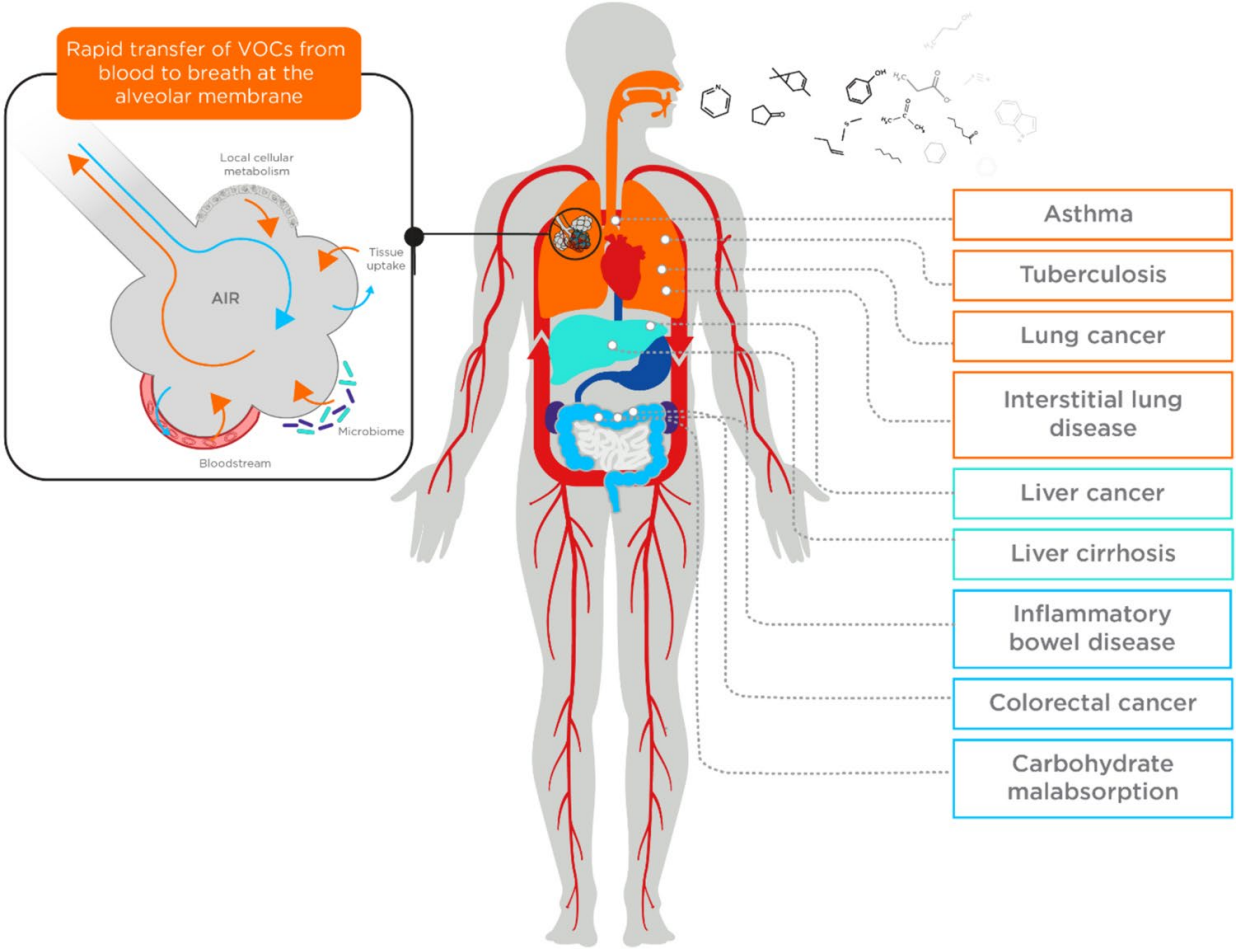
A summary of the mechanism through which VOCs originating from within the body end up in the breath, and some example disease areas that breath VOC analysis has been used for biomarker discovery

Bodily fluids such as blood contain cells, proteins, lipids, and metabolites that can be measured as biomarkers to inform on underlying physiological processes for various clinical purposes. The volatile organic compounds (VOCs) contained within bodily fluids are becoming increasingly studied throughout the medical field for their potential to act as useful biomarkers due to their unique properties (Fig. 1). VOCs contain at least one carbon atom and are gaseous at room temperature and standard pressure conditions. This means that VOCs generated by human metabolism (endogenous) can cross most biological membranes away from their point of origin, and be emitted from bodily fluids including breath, urine, and feces (Amann et al., 2014; Bax et al., 2019; Drabińska et al., 2021). VOCs can also enter the body from external sources such as diet, microbial metabolism, prescription drugs, and environmental exposure (exogenous). Hundreds of VOCs detected from the human body have now been reported, with over 2800 VOCs assigned from breath (1488), skin secretions (623), feces (443), saliva (549), urine (444), milk (290), semen (196) and blood (379) (Drabińska et al., 2021). VOCs can be derived from numerous metabolic processes such as carbohydrate and lipid metabolism, oxidative stress, enzyme activity, and aerobic and anaerobic fermentation processes of bacteria in the gut microbiome. For example, studies show that lipid peroxidation has been associated with many inflammatory diseases all over the body (Amann et al., 2014; Calenic et al., 2015; Drabińska et al., 2021), and due to the different lipid compositions and redox enzyme complements of different cell types, a unique set of VOC lipid peroxidation products could be specific for different disease pathophysiology (Rahman & Kelly, 2003). The detection, identification, and quantification of VOCs can provide a different view into the processes of the body than can be gained from protein and nucleic acid analysis. Discovering the normal, and pathophysiological mechanisms that underlie the production of VOCs may support the translation of new VOC biomarkers, or provide novel insights into new therapeutic approaches for treatments of various diseases.

Many current diagnostic methods are limited by accessibility, patient discomfort, repeat sample collection in a given period (as with fecal sampling, urine, or blood), usability, and cost. One of the major benefits of utilizing VOCs is that they can be sampled non-invasively from exhaled breath. VOC analysis could therefore address currently unmet clinical needs to improve public health, including early disease diagnosis. Breath VOC analysis has been investigated as a more attractive early screening tool for cancer (Hanna et al., 2019; Janfaza et al., 2019) due to its non-invasiveness, ease of repeat sampling, generally low cost, and accessibility, especially as breath can be collected at home. This includes hydrogen and methane breath tests (HMBTs) that are available as at-home test kits to diagnose certain gastrointestinal conditions. Despite the promise of exhaled breath VOC analysis, several ongoing methodological challenges have limited the translation of VOC biomarkers into the clinic. This includes a lack of standardized methodologies for breath collection and analysis workflows that ensure that breath VOC data are reproducible.

This review aims to critically evaluate the current state of the literature on the utility of VOCs in exhaled breath as disease biomarkers, in both clinical and research settings. In the first section, methodological challenges will be outlined, including the issue of standardization which involves the reliability and repeatability of breath VOC data, along with the recent progress made to address and overcome them. Next, we present recent key papers published since 2020 in specific disease contexts, and evaluated these in the context of historical literature. Several key papers that have significantly advanced the field have been published before this time point, however, there has already been significant discussion of these publications in previous reviews (Gashimova et al., 2022; Haworth et al., 2022; Ibrahim et al., 2021; Issitt et al., 2022b; Metwaly et al., 2023; Murgia et al., 2021; van der Sar et al., 2023; van Vorstenbosch et al., 2023a; Wang & Davis, 2024; Westphal et al., 2022). Therefore, we sought to instead discuss more recent papers, and where appropriate, highlight earlier publications to put progress into context. Respiratory disease, infectious disease, gastrointestinal disease, and liver disease are included. Cancer is a key field that breath VOC research shows strong promise, and specific papers will be discussed within relevant disease areas (i.e. lung cancer within respiratory disease). Finally, the application of VOC biomarkers for drug development will be presented. By highlighting methodological considerations and the progress in VOC breath science since 2020, this review provides insights into what future directions are needed to fast-track VOC biomarker discovery, validation, and ultimately translation into the clinic.

## Breath research methodology

### The properties of breath

Exhaled breath has a unique property in that it is comprised of air that has been inhaled immediately prior from the environment, as well as air that was already in the lungs. The breath can be separated into different fractions, including dead space air that does not participate in gas exchange with the blood, and alveolar air, which is air deeper in the lungs that is in direct contact with the alveolar membrane—the site of gas exchange. It is this alveolar air which VOCs from the blood are exchanged into, and so collection methodologies that can selectively collect from the deeper, alveolar fraction of breath are advantageous (Gashimova et al., 2022; Miekisch et al., 2008). This will maximize the collection of endogenous VOCs, and exogenous VOCs that enter the breath through blood-alveolar exchange. There is a negligible amount of air deep in the lungs that does not take part in gas exchange in healthy individuals (alveolar dead space air), but it is important to note that this will increase in proportion in certain respiratory disease states.

It is important to differentiate between a contaminating VOC inhaled immediately before breath sampling from the surrounding background air (henceforth referred to as background VOCs), and exogenously derived VOCs that have originally been introduced into the body via external sources such as diet and the microbiome. It was previously thought that exogenous compounds were very unlikely to bear much information related to underlying biology on principle, however, this is now well understood to not be the case. For example, VOCs present in breath derived from the microbiome such as the short-chain fatty acids (SCFAs) are exogenous but can interact dynamically with human biological processes, and therefore can have significant roles in the maintenance of health and development of disease (den Besten et al., 2013; Lee & Zhu, 2021; Parada Venegas et al., 2019). There is also a grey area in which VOCs that have been continually inhaled from the environment previously over longer periods can enter the body to interact with underlying physiology. They can be excreted throughout time in exhaled breath (such is the case with exposure-derived VOCs from sources such as pollution), and so still can be informative (Pleil & Stiegel, 2013).

## Breath collection methodology

### Off-line approaches

The complexity of breath as a matrix, containing hundreds, if not thousands of VOCs (Drabińska et al., 2021), means that studies usually rely on techniques like Gas Chromatography-Mass Spectrometry (GC–MS) as the gold standard due to its heightened sensitivity and capacity to handle diverse VOCs and low concentrations. This requires the collection and storage of breath for transportation to a laboratory for analysis (off-line). However, contamination from background VOCs can be easily introduced during breath collection and sample storage depending on the choice of offline analysis methodology. Tedlar®, Nalophan^®^, and Mylar^®^ bags are a common method of breath collection for offline techniques, whereby participants in breath studies exhale and fill the bags (Ghimenti et al., 2015; Mager et al., 2024; Sola-Martínez et al., 2023). While inexpensive, disposable, and easy to use, studies have shown that these bags can be a source of contaminating compounds like N,N-dimethylacetamide and phenol, and may leak, especially during handling (Beauchamp et al., 2008; Ghimenti et al., 2015; Groves & Zellers, 1996; Harshman et al., 2020; Steeghs et al., 2007; Sulyok et al., 2001). Exhaled breath also contains water vapor, which is a major contributor to the degradation of volatile compounds contained within breath over time in storage. It is possible to reduce the contamination of bags and storage effects from bags by transferring the collected breath to thermal desorption tubes (Brinkman et al., 2017, 2020). However, the majority of the breath sampled in this manner will contain dead space air with a minority of alveolar air, which can result in the introduction of many background VOCs, and make quantifying low-level breath-bourne VOCs challenging (Gashimova et al., 2022).

Another common method to collect breath is through the Bio-VOC™ breath sampler, which is lightweight easy to use, collects alveolar air that can be transferred to a sorbent tube, with a disposable mouthpiece (Dyne et al., 1997). This device has been used in many breath studies to identify breath biomarkers of diseases such as liver disease (Dadamio et al., 2012; Van den Velde et al., 2008), COPD (Phillips et al., 2012), and cancer (Patterson et al., 2011; Poli et al., 2010), however, it only collects a small volume of breath, and therefore may not be suitable to reliably detect low-concentration VOCs (Kwak et al., 2014). The ReCIVA^®^ Breath Sampler was developed by a broad consortium of breath researchers and engineers and is another potential methodology for breath collection (Kitchen et al., 2015). The device enables the simultaneous collection of replicate breath samples by directly capturing and pre-concentrating VOCs from breath onto multiple sorbent tubes. Moreover, it is connected to the CASPER^®^ Portable Air Supply to better standardize the inhaled air of breath test participants. The built-in pressure sensors adapt to the breath patterns of each subject, and activate the pumps to draw air from specific breath fractions, i.e. alveolar air. The utility of the ReCIVA device for breath collection has been independently evaluated as a practical and precise method, capable of obtaining comparable results to breath collection in bags whilst eliminating the common pitfalls and supporting robust breath sampling across multiple sites (Azim et al., 2019; Harshman et al., 2020). However, the device is expensive, and therefore is not as cost-effective as cheaper options like bags. The consumables used with the ReCIVA (such as disposable masks) must also be stored correctly before use to reduce contamination from siloxane-based VOCs (Holden et al., 2020).

### On-line approaches

Contrary to off-line approaches that have already been discussed, “on-line” breath sampling involves the direct introduction of breath into an analysis device. On-line analyzers involve directly sampling the breath at the point of analysis (such as in real-time breath analyzer devices), and therefore avoid many sampling and storage-related artifacts of off-line methods. These include Secondary Electro-Spray Ionization (SESI), Selected Ion Flow Tube Mass Spectrometry (SIFT-MS) (which can be used with off-line breath collection also), and sensor-based approaches such as eNoses. However, there are currently no on-line techniques that can provide the same level of signal-to-noise and sensitivity as off-line techniques.

There is a preference for the development of portable, point-of-care (PoC) devices capable of analyzing the breath of patients rapidly, without the need to transport samples to the laboratory (Wang & Davis, 2024). However, the development of such tests relies upon a solid understanding of the relevant compounds in breath, the mechanisms contributing to their presence, and the connection between their concentration to the specific disease. To ascertain this, repeatable chemical identification of breath compounds must occur, which is rarely possible from sensor-based approaches. The sensor arrays from eNoses respond to different chemical groups and detect the pattern of breath, or “breath print” rather than identifying individual compounds. Sensor array eNoses have been used to study breath composition for many years (Das & Pal, 2020; van der Sar et al., 2021). Although promising, the data alone cannot provide insight into the disease mechanisms. More often, device-specific data can be challenging to translate to other devices or technologies. Therefore, establishing the baseline of breath composition, crucial for developing portable devices, currently relies on offline techniques like GC–MS to discover and validate specific breath biomarkers using top analytical techniques. Fundamental, foundational research like this needs to come first to then expedite the development and translation of portable devices.

### Standardization of breath collection

With these different methodologies possible, it is critical that the results across different techniques are repeatable, or that there is an understanding of the impact that different sampling methodologies may have on the result (i.e. specific VOCs associated with bags). It must be strongly noted that different breath collection methodologies will suit different clinical contexts and potential applications. This is especially relevant considering the potential on-line and off-line workflows, as both have their strengths and limitations that better suit them for different clinical scenarios. Therefore, it is important to better understand how different methodologies may impact breath results, rather than advocating for the use of a single collection methodology to improve standardization. A greater understanding of normal human breath, as well as reference databases would allow for different breath collection platforms to be compared against and optimized, enabling benchmarking, and the creation of instrument controls.

## Breath analysis methodology

### Background contaminating VOCs

Previous work has estimated that background contaminants could account for more than 70% of the exhaled breath signal in their study (Harshman et al., 2020), highlighting the need for robust, standardized statistical procedures to address this key challenge for breath researchers. In the past, background samples have not been collected alongside breath samples, and simple calculations utilizing peak area of VOC abundance have been used to determine breath-bourne VOCs (Nowak et al., 2021; Sharma et al., 2021; Sukaram et al., 2023). To ensure accurate analysis, breath studies should include background samples collected alongside breath samples at the same site for more rigorous calculation of breath vs background signals. This study design can be incorporated with any breath sampling methodology and quantitative analysis workflow.

Background correction techniques can be undertaken via the calculation of an alveolar gradient that directly compares signal strength in breath to background samples, simultaneous monitoring of inhaled and exhaled breath fractions, and using a lung washout with synthetic air to identify background VOCs (Hewitt et al., 2022; Maurer et al., 2014; Phillips, 1997; Schubert et al., 2005; Spaněl et al., 2013; Westhoff et al., 2022). A common method is collecting the ambient air in the same location where breath sampling is being performed, but this will not sample VOCs that originate from the sampling equipment. This is an important contributor to background VOCs, and specific VOCs can be emitted by different components of the sampling apparatus (Pham et al., 2023). Since VOCs can be introduced at multiple points throughout sample collection, and analytical process, it is important to ensure that both breath and background samples undergo comparable collection and handling processes (Di Gilio et al., 2020).

Quantitative metrics can be used to analyze which volatiles are enriched in the breath in comparison to the background, and different thresholds can be applied to the data to accommodate different contexts. These quantitative metrics can be based on how frequently VOCs show in the breath of a population at a threshold concentration (i.e. at least 50% of a population should have a selected number of standard deviations above the background sample concentration). This approach has been used during the development of the VOC Atlas, an ongoing project to develop a reference range of VOCs present in the breath that are able to be distinguished from background based on tiers of quantitative metrics (Hatch et al., n.d.). Due to how important this is to advance breath research, many groups both academic and industrial are also developing reference databases of normal human breath (Chou et al., 2024b; Human Breath Atlas, 2024). Several reference databases from multiple sources and groups across the world and global collaboration in these efforts will allow for comparison between databases for even greater confidence in what the normal ranges of VOCs in the breath are.

## Developing highly sensitive and specific VOC biomarkers

One major area that requires careful consideration is whether candidate biomarkers identified through statistical models between patients and healthy controls can be translated into more clinically relevant contexts. This is especially important regarding the ability to maintain adequate specificity and sensitivity in clinical settings where breath tests are given to populations with most likely similar conditions. As VOCs are reflective of metabolic pathways, it is expected that patterns of VOCs are highly individual, with certain VOCs being shown to fluctuate depending on the time of day in those with asthma (Wilkinson et al., 2019). With larger variation expected in the data, large sample sizes and high statistical power are required to gain confidence in data, driving up the costs of clinical trials. Given the low concentrations of VOCs in breath and potential low signal-to-noise challenges, achieving high sensitivity and specificity at the early stages of clinical trials is crucial. Without this, breath tests are unlikely to maintain clinically useful accuracy when deployed in real-life clinical settings. Approaches to boost signal-to-noise such as exogenous VOC (EVOC^®^) probes (discussed in later sections) are one approach that can help to improve the sensitivity and specificity of breath tests (2023b; Ferrandino et al., 2020; Gaude et al., 2019; Labuschagne et al., 2022).

**Fig. 2:**
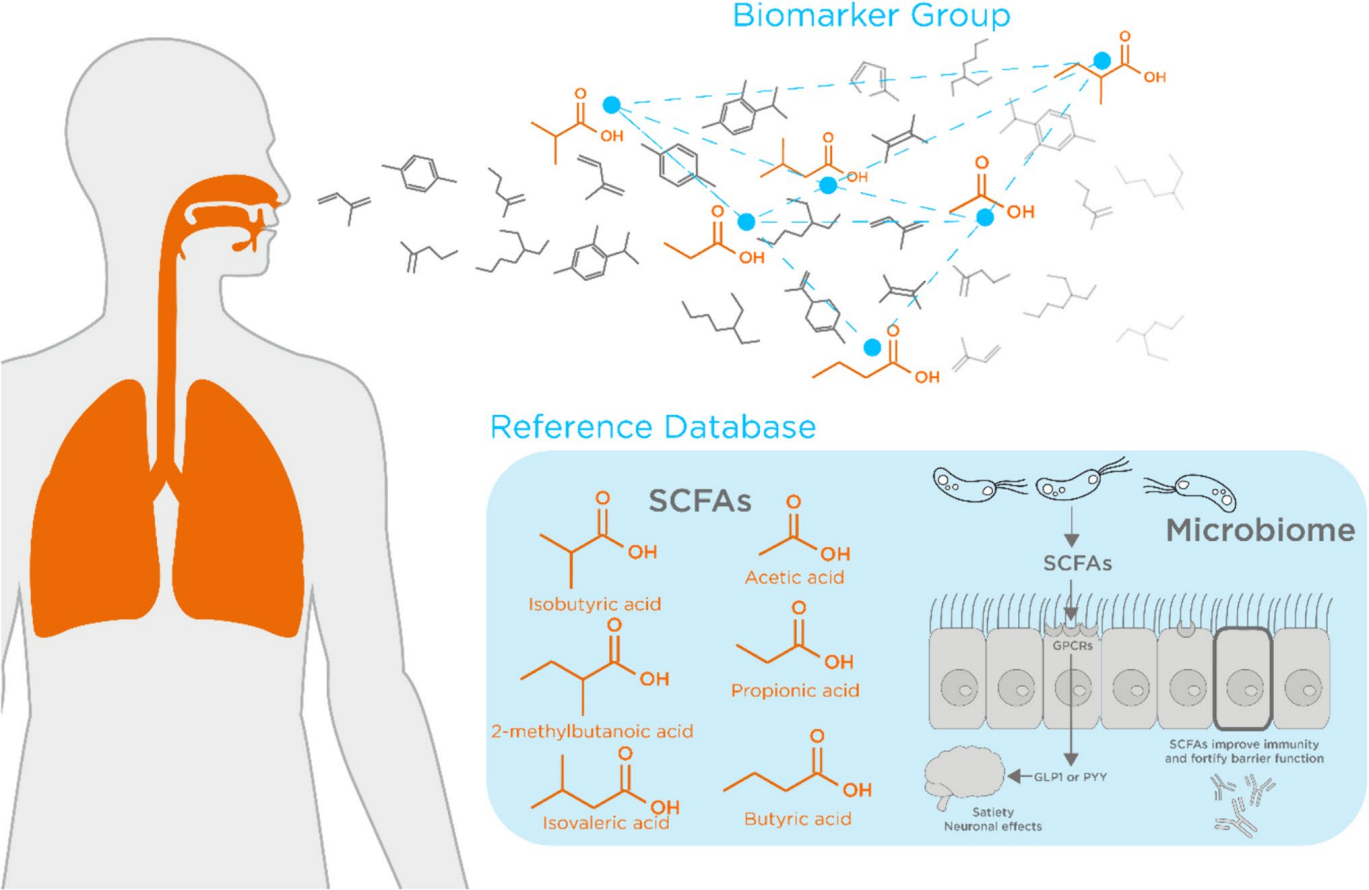
A hypothetical schematic indicating how complex analysis of biomarkers in context to each other in the breath, alongside reference databases can enhance the power of breath research for innovative tools for clinical use

Another approach is to use more complex analytical workflows. Machine learning approaches are increasingly being used to analyze breath VOCs, aiming to identify patterns in how relevant compounds cluster together (Haripriya et al., 2023) This can include other clinical data to maximize the usefulness, and interpretation of breath test results, and has been utilized with success previously (Ibrahim et al., 2022). Handling highly complex VOC data in breath is key to identifying meaningful differences between study cohorts, especially if the origin of the identified VOCs in the body is known, and the normal ranges in a healthy population are available in a reference database (Fig. 2). Gaining a deeper understanding of the networks of interactions of VOCs can help to relate them to underlying physiology, and therefore algorithms can learn what are likely to be false-positive hits. Utilizing the patterns of breath data rather than individual VOCs is similar to how e-nose technology operates (Hao & Xu, 2017; Haripriya et al., 2023; Seesaard et al., 2012; Shlomi et al., 2017), with different sensors responding to different chemical groups. However, sensor-based techniques often suffer from a lack of repeatability, as well as technical issues such as drift and sensor faults (Haripriya et al., 2023; Le Maout et al., 2018). The ability to identify specific VOCs rather than just overall patterns makes spotting likely contaminating VOCs that are more likely to be unrelated to underlying biology easier.

### Standardized chemical identification of VOCs

Improving confidence in chemical identification has been a focal point for standardization in breath research, as highlighted by experts during previous breath conferences (Beauchamp et al., 2016; Chou et al., 2024a, 2024b; Schmidt et al., 2021). Untargeted breath biomarker discovery workflows make up most breath VOC studies and often result in a list of tentatively identified VOCs through comparison to publicly available standard libraries, such as those provided by the National Institute of Standards and Technology (NIST). This increases the risk of misidentifications due to differences in methodology and instrumentation between untargeted datasets and reference libraries and can result in lower replicability. Confidently identifying a VOC in a breath sample requires a comparison to purified chemical standards analyzed using the same instrumentation and methods, which is costly and time-consuming (Fiehn et al., 2007; Sumner et al., 2007). It is important to utilize platforms that have high mass accuracy and resolution capability which can provide precise exact mass measurement of ions and separation of closely spaced mass peaks. High specificity by accurate determination of molecular formula and the ability to distinguish between different VOCs with similar masses is a key goal to increase the accuracy of VOC identification and therefore replicability across the field of VOC breath research.

Incorporating potential benchmarking protocols such as the Peppermint Initiative, and instating standards from the Metabolomics Standards Initiative (MSI) could produce more quantitative criteria for compound identification pipelines and increase the repeatability of findings (Fiehn et al., 2007); Henderson et al., 2020). There are different levels of MSI confidence in identification, with level 1 being the highest confidence as an “identified compound”, and level 4 being an “unknown compound.” Achieving level 1 or level 2 (putatively annotated compound) indicates that stringent requirements have been met and is a practical goal for breath studies to aim toward. The design of studies can be modified accordingly to best achieve this, including chemical reference standards as part of analytical workflows. Confidently knowing the IDs of VOCs in the breath can significantly help with biological interpretation as to why certain VOCs may be increased or decreased in abundance, which then may help link the findings to specific pathways that may be involved in pathophysiology. Along similar lines, establishing a list of VOCs from healthy individuals is a key step in differentiating between disease states, and is a major pursuit of many breath researchers (Chou et al., 2024b; Human Breath Atlas, 2024; Sasiene et al., 2024). This will notably contribute to a better understanding of the mechanistic origins of breath VOCs in the body, improving the standardization in the field through a quantitative baseline for comparison and benchmarking, and forming the foundation for accelerated breath biomarker discovery studies to the translation into clinical settings.

## Respiratory disease

Breath is a waste product that originates from the respiratory tract, and therefore can contain VOCs directly emitted from the surrounding tissue. Fractional exhaled nitric oxide (FeNo) breath tests measure exhaled nitric oxide (NO) as a marker of airway inflammation for the assessment of asthma and are currently used in the clinic. However, there is a need for better biomarkers to improve the accuracy of this test, as well as additional breath tests for a wider number of respiratory conditions. Previously, analyzing the VOC content of breath was shown to be able to discriminate healthy controls from patients suffering respiratory diseases including lung cancer, chronic obstructive pulmonary disease (COPD), and asthma, and so there is hope for a broader range of breath tests to become available in the near future (Dallinga et al., 2010; Koureas et al., 2020; Monedeiro et al., 2021; Ratiu et al., 2021; Van Berkel et al., 2010; van der Kamp et al., 2021).

A major recent study conducted by the EMBER consortium analyzed breath from subjects visiting the emergency department due to breathlessness (n = 277) caused by various conditions such as severe exacerbation of asthma, COPD, heart failure, and pneumonia, and compared them to healthy controls (Ibrahim et al., 2022). A particular strength of this analysis was the adherence to MSI criteria, 58 VOCs were identified against standards (level 1), 21 putative identities were assigned based on mass spectral and retention index library matches (level 2) (Fiehn et al., 2007; Sumner et al., 2007). A machine learning approach uncovered predictive VOC biomarkers for the different conditions, of which 101 identified breath VOCs could be associated with cardiorespiratory patients.

Several classes of exhaled VOCs were also highly correlated with disease groups, as well as subgroups in this study (Ibrahim et al., 2022). As an example, there were eight VOCs associated only with COPD, 12 further associated with COPD, and one other condition. The prevalence of hydrocarbons and carbonyls in this study strongly associates lipid peroxidation as a major origin of characteristic VOCs in the breath of those with respiratory conditions. Chronic inflammation generates oxidative stress, resulting in the generation of reactive oxygen species that can react with unsaturated fatty acids in the cell (lipid peroxidation), and the subsequent release of characteristic volatile compounds such as alkanes, aldehydes, hydrocarbons, and carbonyls (Caldeira et al., 2012; Ibrahim et al., 2022; Monedeiro et al., 2021; Ratcliffe et al., 2020; Shahrokny et al., 2023; Sharma et al., 2021; van Vliet et al., 2017; Wijsman et al., 2024).

It is important to note that chronic respiratory tract inflammation can also occur in non-disease conditions. For example, previous studies have linked exhaustive exercise with signs of damage to the respiratory system, including increased susceptibility to upper respiratory tract infections, and inflammation (Howatson et al., 2010; Robson-Ansley et al., 2012; Webner et al., 2012). A recent study investigated VOC profile changes (n = 24) before and after participants ran an ultra-marathon (Chou et al., 2024a). A total of 63 compounds were significantly different in abundance between the pre- and post-race samples, 12 decreased and 51 increased, and the specific changing VOCs suggest the involvement of lipid peroxidation and inflammation (2-butanone and 4-heptanone), and possible altered gut microbiome activity (acetate, 2,3-butanedione, and 2,3-butanediol) in response to exhaustive exercise (Chou et al., 2024a).

### Asthma

**Fig. 3:**
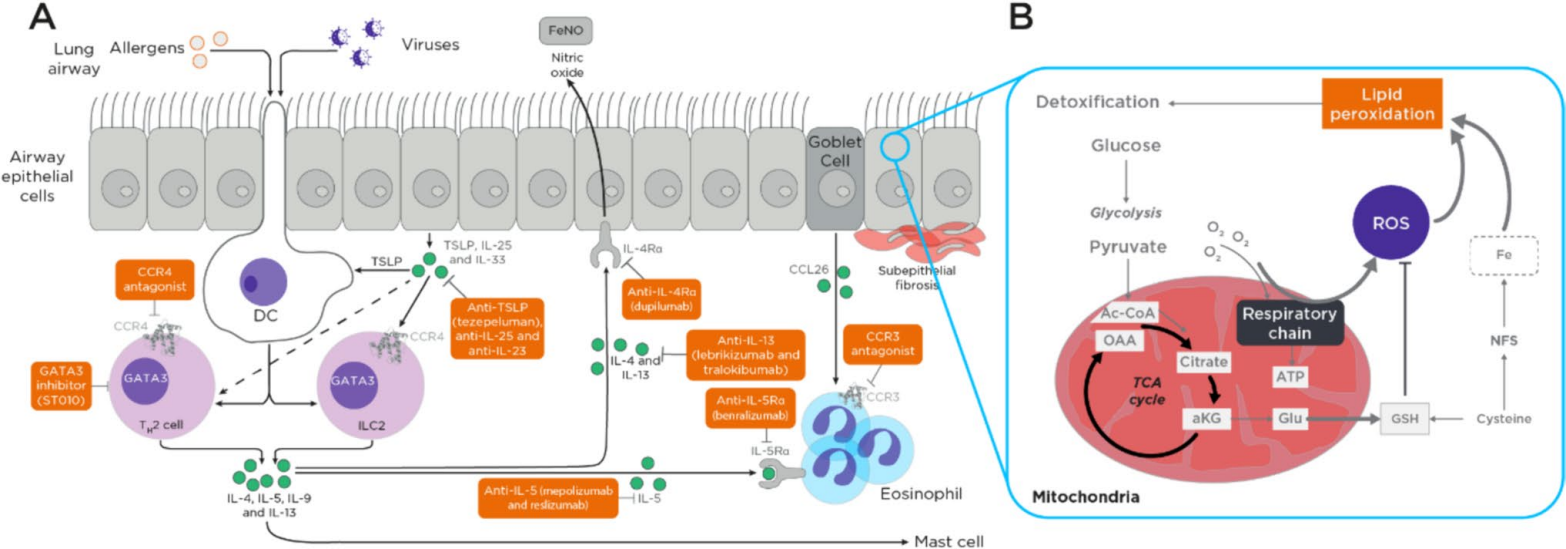
**A** An overview of the pathophysiology involved in asthma producing a breath. as an example of a respiratory disease. **B** Lipid peroxidation is thought to be one of the major producers of VOCs

In asthma, the inflammatory process can be categorized into two major types based on the predominant type of immune cells causing the inflammation: eosinophilic (type 2) or neutrophilic (type 1). Most asthmatic patients have type 2 inflammation. Type 2-low asthma is difficult to define due to the lack of reliable biomarkers, but includes patients with neutrophilic asthma, which is characterized by the absence of eosinophilic inflammation in the airway with excessive neutrophils instead (Hudey et al., 2020; Locksley, 2010). These complex interactions between the immune system, and the respiratory tract are associated with a unique pattern of volatiles in the breath (Fig. 3) (Rahman & Kelly, 2003). Many hypothesized lipid peroxidation VOC products including furan, 2-methyl- and tetrahydrofuran have been shown to change in abundance in the breath of patients with asthma (Ibrahim et al., 2022; Ratcliffe et al., 2020; van Vliet et al., 2017; Vliet et al., 2016; Wijsman et al., 2024).

Distinguishing asthma from other diseases is an important goal for breath VOC biomarkers to meet clinical useability. The breath of women of childbearing age were sampled in a recent study (training set, n = 211; validation set, n = 125) three months after childbirth to test whether VOCs could discriminate between asthmatics with coexisting atopic diseases (A-AD) and non-asthmatics (NA) (with or without atopic diseases) (Sola-Martínez et al., 2021). Acetone, 2-ethyl-1-hexanol, and tetrahydroisoquinoline derivatives together discriminated A-AD and NA with 71% sensitivity and 63% specificity in the validation set. The study couldn’t differentiate between asthmatics with or without coexisting atopic diseases because all but one subject was diagnosed with other atopic diseases. However, this study demonstrated that breath VOCs are a feasible tool to help distinguish asthma patients from those with other atopic diseases (Sola-Martínez et al., 2021).

A large number of VOCs capable of distinguishing children with allergic asthma (n = 48) from healthy controls (n = 56) were recently identified using on-line breath analysis (SESI/HRMS), and were able to generate an AUC of 0.83 (Weber et al., 2023). VOCs associated with asthma such as succinate and glutarate suggested an increase in lysine metabolism, which aligns with some previous data (Carraro et al., 2018). However, the authors note the limitation of only moderate confidence in the putative identification of VOCs due to the lack of chromatographic separation in on-line SESI/HRMS, and software utilized that uses computational analysis to determine chemical structures through identifying potentially matching fragmentation patterns (Dührkop et al., 2019; Weber et al., 2023).

Breath samples (n = 100) from the SysPharmPediA cohort of children with asthma were analyzed in a recent study to identify candidate VOC biomarkers capable of distinguishing between controlled and uncontrolled asthma (Khamas et al., 2024). Acetophenone, ethylbenzene, and styrene were identified, and were validated in two independent cohorts (U-BIOPRED and PANDA) (Khamas et al., 2024). A machine learning tool sPLS-DA resulted in an AUROCC of 0.83 (95% CI 0.65–1.00) for the training set, and an AUROCC of 0.77 (95% CI 0.58–0.96) with an accuracy of 0.88, a sensitivity of 0.90, and a specificity of 0.82 for the test set, demonstrating the potential power of VOC biomarkers for asthma. Importantly, these candidate VOC biomarkers overlap with previous literature that also analyzed the exhaled breath of those with asthma (Caldeira et al., 2012; Gahleitner et al., 2013; van de Kant et al., 2013; van der Kamp et al., 2021).

A very recent study by Djukanović et al. (n = 191) used a multi-omics approach to identify candidate predictive biomarkers of response to omalizumab (Djukanović et al., 2024). Amongst the sampling mediums analyzed, breath and plasma demonstrated the greatest potential in generating biomarker panels with high accuracy and potential translatability to the clinic, with two putative panels emerging from VOC breath analysis, and one from plasma metabolomics. The two breath panels were benzothiazole, acetophenone, 2-pentylfuran, methylene chloride, and 2-methylbutane: ROCAUC of 0.835, and 2-ethyl-1-hexanol, toluene, 2-pentene, nonanal, and a VOC of unknown identity, which could predict ≥ 50% exacerbation reduction with an ROCAUC of 0.780. Interestingly, GC–MS breath VOC analysis was shown to outperform eNOSE technology. Although lipids had greater predictive ability than breath VOC biomarkers, the authors considered breath VOC analysis to be a superior biomarker discovery and analytical platform due to the ease of breath collection, and an easier development pathway of point-of-care devices for use in a clinical setting (Djukanović et al., 2024).

### ILD

Interstitial lung diseases (ILD) are a group of over 200 similar chronic lung conditions, the most common and lethal of which is idiopathic pulmonary fibrosis (IPF) (Wijsenbeek et al., 2022). ILD has been studied using breath with promising results, but there is currently a lack of large validation studies to progress the findings into a clinically useful test (van der Sar et al., 2023). A recent study (n = 157) analyzed the breath of subjects diagnosed with IPF, connective tissue disease-associated interstitial lung disease (CTD-ILD), and controls (Plantier et al., 2022). In this study, 34 VOCs were identified that could distinguish IPF patients from control patients (84.6% accuracy), and a subset of 16 VOCs could distinguish IPF from CTD-ILD (accuracy 76.9%). The putatively identified VOCs that contributed the most to the differences between IPF and controls were benzaldehyde which increased in IPF patient’s breath, and ethanol, heptane, dimethyl sulfide, and an unknown compound that all decreased in IPF patient’s breath. A key observation in this study was that dimethyl sulfide decreased in IPF whereas the levels of dimethyl sulfone increased. Dimethyl sulfone is formed through the oxidation of dimethyl sulfide by hydrogen peroxide, and hydrogen peroxide has previously been shown to be produced in significantly higher amounts in IPF through the activation of NADPH oxidase 4 by transforming growth factor β (TGFβ) (Hecker et al., 2009). The authors posit this mechanism as a potential link between IPF pathophysiology and VOC content of exhaled breath. The VOCs 2-heptanone and 4-pentan-1-ol were decreased in the breath of CTD-ILD patients compared to IPF, and healthy controls, indicating that these two VOCs might be specifically related to CTD-ILD pathophysiology. Other work has emphasized the role of 11 carbonyl VOCs in breath and demonstrated the potential of measuring these compounds alongside pulmonary function tests to distinguish ILD subtypes and classify the severity of disease burden (Taylor et al., 2024). However, none of these VOCs appeared to be shared between the VOCs that could distinguish IPF and CTD-ILD in the study by Plantier et al., so the use of different breath collection methodologies and analytical procedures may be significantly impacting the results (Plantier et al., 2022).

### Lung cancer

A review focused on breath VOC profiles in lung cancer highlighted that different sample collection procedures, differences in ambient air in different sampling locations, heterogeneity in patient biology, and analysis methodology used are important factors contributing to highly heterogeneous results (Wang et al., 2022). Recent papers have shown progress in this area using both off-line and on-line (Janssens et al., 2020; Kort et al., 2020; Koureas et al., 2020), and a recent review reported 34 VOC candidate biomarkers that have been reported in more than five studies (Schmidt et al., 2023). In particular, the process of lipid peroxidation producing specific aldehydes, especially pentanal, hexanal, and heptanal, have been raised as repeatably represented across the literature in breath VOC profiles of those with lung cancer (Sutaria et al., 2022). However, despite the large amount of work in this area, there are currently no clinically validated breath biomarkers or tests suitable for lung cancer screening due to historical issues with standardization (Antoniou et al., 2019; Choueiry et al., 2022; Keogh & Riches, 2022).

An innovative study design aimed to identify breath biomarkers of lung cancer through perioperative dynamic breath testing, whereby the breath of 84 patients with lung cancer was analyzed before, and after surgery to remove the tumor (Wang et al., 2022). This study design allows for a more confident establishment of potential cause-and-effect relationships between breath VOC changes and lung cancer presence. A similar approach has been used for the discovery of breath biomarkers of liver disease to great success, and so demonstrates a powerful method of increasing the confidence of identified breath biomarkers across other studies (Fernández del Río et al., 2015). This study showed that 16 VOCs significantly changed in abundance in the breath after surgery as candidate lung cancer biomarkers, 15 VOCs showed a significant reduction: 2-hydroxyacetaldehyde, isoprene, pentanal, butyric acid, toluene, 2,5-dimethylfuran, cyclohexanone, hexanal, heptanal, acetophenone, propylcyclohexane, octanal, nonanal, decanal, and 2,2-dimethyldecane, and acetaldehyde showed a significant increase after surgery (Wang et al., 2022). A validation study was also conducted with a comparatively large number of participants (n = 157 lung cancer patients, n = 368 healthy controls), and all 16 VOCs previously identified were significantly increased in the patients with lung cancer compared to controls. As cytochrome P450 oxidoreductases (PORs) can decarboxylate and desaturate hydroxylated fatty acids and regulate the peroxidation of polyunsaturated fatty acids (Munro et al., 2018; Zou et al., 2020), the authors speculate that CYP450 isozymes and fatty acid types affected by cancer may influence the VOCs in exhaled breath. Alteration in fatty acid metabolism is widely observed in many types of cancer cells, this pattern of VOCs could be markers of cancer in general. (Hoy et al., 2021; Koundouros & Poulogiannis, 2020).

To address whether VOCs could distinguish lung cancer specifically, one study analyzed the VOCs in the breath using GC–MS of those with lung cancer (n = 85) and other types of cancer: esophageal (n = 11), breast (n = 22), colorectal (n = 16), kidney (n = 14), stomach (n = 7), prostate (n = 6), cervix (n = 5), and skin (n = 4) (Gashimova et al., 2023). The VOCs hexane, acetonitrile, 1-methyltiopropene, 1-methylthiopropane, and dimethyl sulfide were associated with lung cancer vs other cancer types, which have been associated with lung cancer previously (Rudnicka et al., 2014; Sakumura et al., 2017; Temerdashev et al., 2023). Breath VOCs were able to distinguish lung cancer and patients with other cancers with moderate sensitivity (68%) and specificity (69%). The accuracy obtained for distinguishing the different types of cancer from each other was poor, however, the authors note the small sample sizes of each group limits the interpretation of the results (Gashimova et al., 2023).

In terms of discriminating lung cancer from other respiratory conditions, 29 candidate VOC biomarkers previously identified in the literature were analyzed in the breath of those with lung cancer, COPD, and asthma (Monedeiro et al., 2021). A broader variety of VOCs were associated with Lung cancer and COPD profiles compared to asthma profiles, with an increased amount of hydrocarbons in lung cancer patients. In lung cancer VOC breath profiles, 41 discriminating VOCs were verified, however, 92% of the compounds associated with lung cancer were also observed in healthy controls. This suggests that observing the breadth of the variety of VOCs in the breath is an important discriminating factor, not just the abundance of specific VOCs. To identify biomarkers and put them to clinical use, untargeted analysis is more suited to gain oversight of the entire VOC profile change between different disease cohorts. Alongside reliable breath VOC reference databases, this could substantially fast-track the discovery, narrowing down, and validation of candidate biomarkers (Chou et al., 2024b; Hatch et al., 2023).

## Infectious diseases

**Fig. 4:**
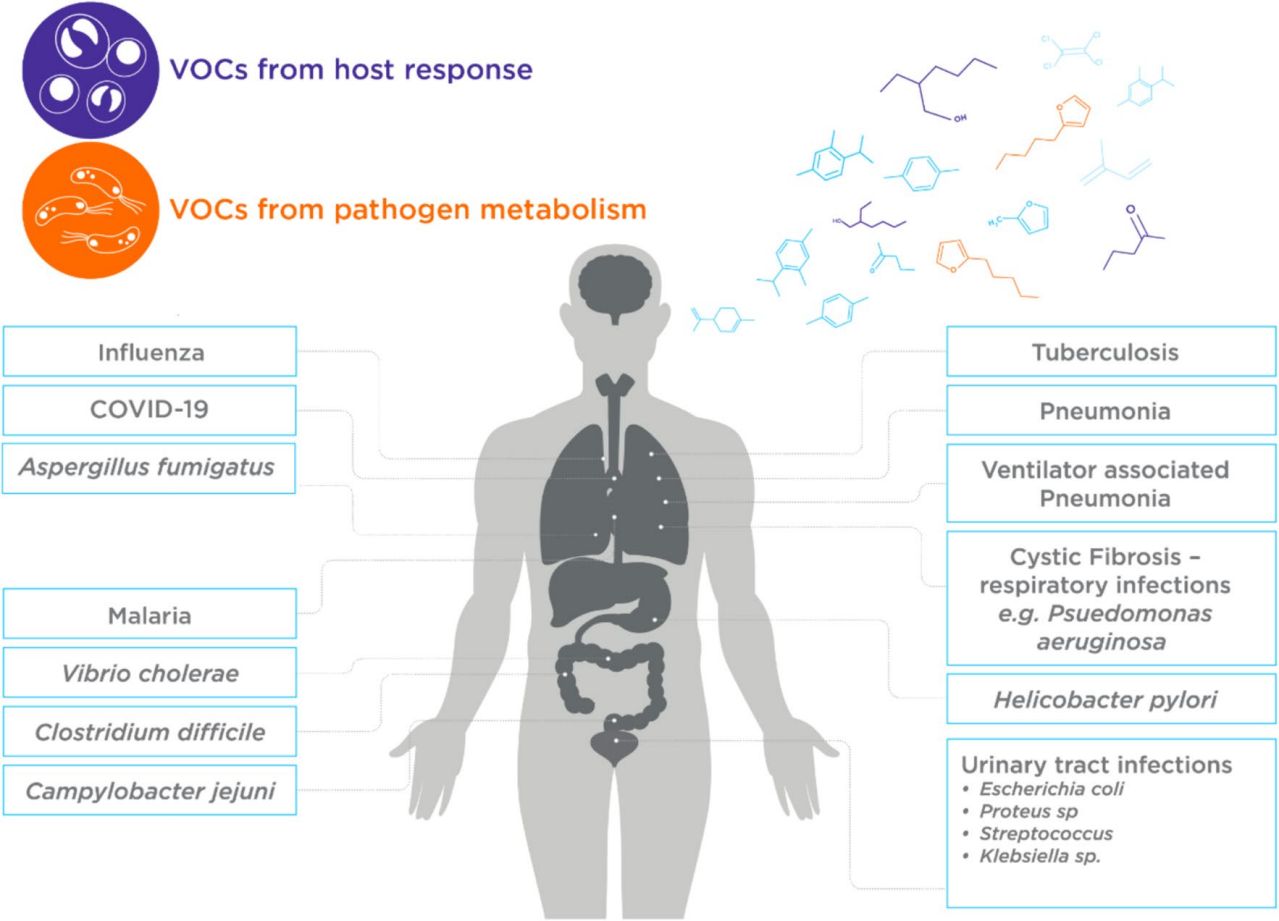
A summary of different infectious diseases that have been studied using VOCs. VOCs in the breath associated with infectious disease can originate either from pathogen metabolism, or VOCs from the host response

The application of breath analysis in clinical settings for infectious diseases has significant advantages for rapid diagnosis, transmission tracking, and management of infectious diseases. For example, the detection of *Helicobacter pylori* (*H. pylori*) infection using a ^13^C-urea breath test is rapid, non-invasive, and highly accurate. The rationale behind using breath tests for infectious diseases is that pathogens can alter the VOC composition of breath either through their metabolism or by affecting host metabolism via the immune response (Fig. 4). An example of the former is the VOC 2-pentyl furan, produced exclusively by fungal species like *Aspergillus fumigatus*: the most common species responsible for invasive aspergillosis (Syhre et al., 2008). Unlike bacterial and fungal pathogens, viruses do not have their own metabolism and therefore do not emit VOCs. Instead, they induce metabolic changes in the host, leading to altered VOC profiles. Therefore, VOCs identified from viral infection should solely reflect changes in host metabolic processes.

### Viral infectious diseases

Interest in breath VOCs for diagnostics of viral infection has surged since the SARS-CoV-2 pandemic. One study (n = 98) showed that aldehydes (ethanal, octanal), ketones (acetone, butanone), and methanol can distinguish hospitalized COVID-19 patients from individuals with other respiratory and cardiac conditions (Ruszkiewicz et al., 2020). Similarly, a targeted analysis (n = 26) involving children infected with SARS-CoV-2 revealed significantly increased levels of aldehydes (octanal, heptanal, nonanal), alkanes (decane, tridecane) and 2-pentylfuran (Berna et al., 2021). This study’s validation in an independent cohort (n = 26) showed that these six biomarkers combined could distinguish infected children with 91% sensitivity and 75% specificity (Berna et al., 2021). A recent study (n = 108) identified 11 VOCs capable of distinguishing mild COVID-19 patients from asymptomatic controls, validated in an independent cohort (n = 40) (Myers et al., 2023). Another recent study (n = 104) suggested that the COVID-19 vaccine enhances enzymatic activity and microbial metabolism in the liver, lung, and gut (Cen et al., 2023).

Compared to SARS-CoV-2, influenza VOC studies before 2020 primarily used i*n vitro* cell cultures rather than clinical breath samples (Phillips et al., 2010; Purcaro et al., 2018; Traxler et al., 2019). This may be because of less urgent clinical need due to the availability of influenza vaccines, or the difficulty to recruit subjects without being confounded by influenza vaccination. However, utilizing breath VOCs to distinguish influenza from influenza-like illnesses could provide significant clinical benefits, especially in the post-COVID era. A recent study (n = 235) demonstrated that four breath biomarkers, mostly n-alkane derivatives, could distinguish influenza from those with similar illnesses with 78% accuracy (Danaher et al., 2022). While promising, the tentatively identified compounds require reference standards for confirmation, and the findings require an independent cohort for validation.

Viral infection can exacerbate existing respiratory diseases. Studies have indicated that cultured airway epithelial cells infected with bacterial and viral pathogens produce unique patterns of VOCs (Abd El Qader et al., 2015; Allardyce et al., 2006; Schivo et al., 2014; Scotter et al., 2006). A key study identified a viral-specific VOC pattern in infected cultured airway epithelial cells, in the breath of healthy volunteers (n = 11) infected with rhinovirus (RV)-A16, and those with COPD exacerbations caused by viruses (Kamal et al., 2021). The specific VOCs included long-chain alkanes like 4,7-dimethyl-undecane, hexadecane, and 2,9-Dimethyl-undecane, with decane levels increasing in both in vitro cultures and in virus-infected healthy volunteers. Similar results were found in the breath of COPD subjects (n = 139) during naturally occurring viral exacerbations, highlighting the potential of VOCs as biomarkers of viral infection (Kamal et al., 2021). Further work is needed to confirm the presence of these VOCs across other breath collection and analysis procedures and to elucidate the mechanisms leading to their production in response to viral infection.

### Bacterial infectious diseases

Like viral infection, bacterial infection can also exacerbate existing respiratory diseases. For example*, Pseudomonas aeruginosa* is a major cause of exacerbations in cystic fibrosis (CF) patients. Despite the high potential of breath-based diagnosis for detecting *P. aeruginosa* through its volatile compounds, translating the findings of potential biomarkers from bacterial culture to clinical settings remains a challenge (Bean et al., 2016; Goeminne et al., 2012; Robroeks et al., 2010; Savelev et al., 2011; Scott-Thomas et al., 2010, 2011; Zscheppank et al., 2014, p. 2). One recent study reviewed 56 VOCs from the literature and evaluated them in a cross-sectional study (n = 53) comprising CF subjects with or without *P. aeruginosa* infection (Kos et al., 2022). The study separated children (n = 25) and adults into different cohorts for breath VOC analysis. Of the 13 detectable breath compounds, three (2-butanone, ethyl acetate, 2,4-dimethylheptane) significantly differed between infected and non-infected children, but not in adults. This highlights the translation challenges between bacterial cell culture and human breath, and the importance of standardization in breath research for reproducibility.

Distinguishing host response compounds from bacterial metabolism in breath is another challenge, as suggested in earlier tuberculosis (TB) studies (Beccaria et al., 2018a, 2018b; Beccaria et al., 2018a, 2018b; Kolk et al., 2012; Küntzel et al., 2018; Saktiawati et al., 2019). *Mycobacterium tuberculosis* (Mtb) infection, which causes TB, remains difficult to diagnose in children. However, a recent study (n = 31) showed that decane, 4-methyloctane, and two other unconfirmed compounds in exhaled breath could classify children confirmed with TB from those with alternate lower respiratory tract infection (LRTI) with 80% sensitivity and 100% specificity (Bobak et al., 2021). While a small cohort size, the study provides insight as TB is a leading cause of childhood mortality, and adults and children have different breath profiles. Another study (n = 435) utilized breath analysis to distinguish latent tuberculosis infection (LTBI) from controls, the latter comprising active TB (ATB) and healthy controls (Fu et al., 2023). The model showed that breath VOCs could distinguish LTBI from controls with 80.0% sensitivity and 80.8% specificity. These findings demonstrate the potential of early LTBI detection, though the tentatively identified VOCs require confirmation with reference standards.

Breath tests can have broader applications in infectious disease diagnosis beyond the lungs. *Clostridioides difficile* (formerly known as *Clostridium difficile*) infection, a serious cause of antibiotic-associated diarrhea, can often be acquired in healthcare facilities. While VOCs from stool samples can detect *C. difficile* infection, stool collection is unpleasant and less convenient than breath sampling (M. Patel et al., 2019; Probert et al., 2004). A recent study (n = 62) demonstrated that breath VOC patterns could predict *C. difficile* infection with high accuracy (AUC = 0.93) (John et al., 2021). Another study (n = 34) showed a panel of 9 VOCs capable of distinguishing infected patients (AUC = 0.72) (John et al., 2024). While these are the first known studies to utilize breath testing for identifying *C. difficile* infection, together they illustrate that VOCs from gastrointestinal infections can be carried via the bloodstream to the lungs and exhaled, supporting the use of breath VOCs for diagnosing various infectious diseases.

### Fungal infectious diseases

Breath VOCs not originating from endogenous metabolic processes can also have great potential as infectious disease biomarkers. For example, the terpenes produced by *Aspergillus fumigatus,* an opportunistic fungal pathogen causing pulmonary aspergillosis in immunosuppressed patients. Several terpenes produced by *A. fumigatus* have been detected in both in vitro culture and breath tests, although not perfectly overlapping (Koo et al., 2014). Interestingly, a recent study (n = 133) showed that exhaled limonene levels, a monoterpene, positively correlated with serum anti-*A. fumigatus* IgG antibody titers in subjects with chronic pulmonary aspergillosis and declined after antifungal treatment (Li et al., 2021). Since terpenes are also synthesized in (dietary) plants, moderate fasting or dietary restrictions may be necessary prior to breath sampling to avoid false positive results if terpenes are applied as biomarkers for diagnosis.

Breath analysis can be a great tool for diagnosing infectious diseases. However, it is crucial to understand that the different pathogens growing and the different competition environments between in vitro and within the host may result in different detection of VOCs. Therefore, repeating findings of the same VOCs from both types of studies is necessary to: (1) confirm the VOC’s origin, (2) understand the mechanisms leading to their presence in breath, and (3) confidently translate these VOCs into clinical biomarkers. Correlational analysis between breath VOCs and clinical metadata or other known biomarkers from other matrices is equally important for understanding the pathways that these VOCs are involved in, which can also facilitate drug development.

## Gastrointestinal disease

**Fig. 5:**
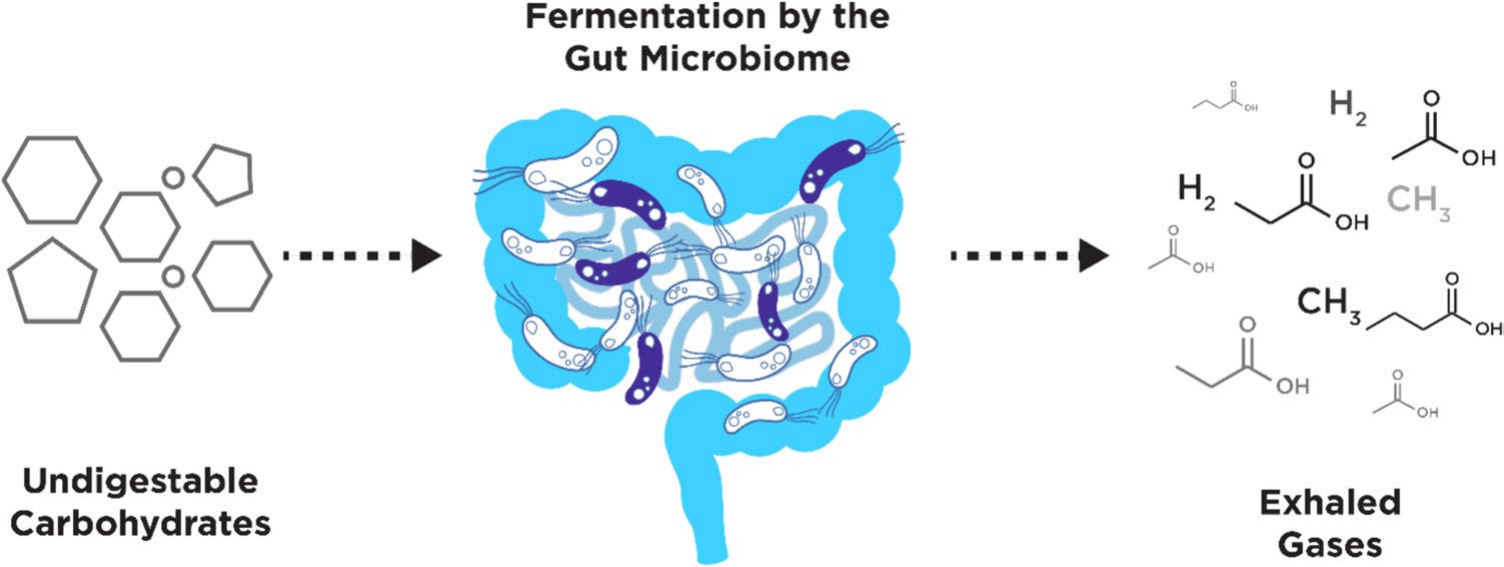
Fermentation of dietary carbohydrates by microbes in the gastrointestinal tract are known to produce key VOCs such as short-chain fatty acids (SCFAs), as well as breath gases hydrogen and methane that are currently used in the clinic to diagnose SIBO and CM

Gastrointestinal (GI) diseases have a high prevalence throughout the globe, with over 40% of people worldwide suffering from a functional gut disorder (Sperber et al., 2021), and 38.4% of all prevalent diseases have a digestive etiology (Wang et al., 2023). *H. pylori* plays a role in chronic gastritis, gastric ulcers, and gastric cancer (Kusters et al., 2006), and is classified by the World Health Organization as a grade 1 carcinogen (Asaka et al., 2001). Hydrogen and methane breath tests (HMBTs) are well-established clinical tools for diagnosing small intestinal bacterial overgrowth (SIBO), intestinal methanogen overgrowth (IMO), and carbohydrate malabsorption (Enko et al., 2014; Lee et al., 2000; Rangan et al., 2022). The fermentation of fibers from the diet is a process that results in the production of SCFAs, which are key metabolites that are thought to impact health (Fig. 5). Hydrogen can be converted by methanogens if present in the gastrointestinal tract into methane, therefore hydrogen levels are interpreted alongside methane for diagnostic purposes (Sachdev & Pimentel, 2013; Triantafyllou et al., 2014). While HMBTs and the carbon-13 breath test are valuable tools for specific GI conditions, VOCs in the breath could address further needs in GI medicine, including differentiating between gastric cancers, irritable bowel syndrome (IBS), and inflammatory bowel disease (IBD). These conditions all present with similar symptoms but have very different treatments and long-term consequences. As gases like hydrogen from the gastrointestinal tract can be detected in exhaled breath within a few minutes of being produced (Read et al., 1985), VOC profiling in exhaled breath can serve as a rapid, reliable, and non-invasive method to assess intestinal health.

### Gastrointestinal cancer

Early-stage cancers are often asymptomatic and difficult to detect. Fecal immunochemical testing (FIT) is the most used non-invasive screening test for most gastrointestinal malignancies, however, a negative result cannot be used to exclude gastric cancers in patients who have persistent GI symptoms (Jones et al., 2023). A recent study (n = 43) investigated the presence of VOCs in exhaled breath associated with gastric cancer and found that nine VOCs differed significantly between the control and cancer patient groups (Jung et al., 2021). This study also found that when participants were divided into control, early gastric cancer, and advanced gastric cancer groups, propanal, acetamide, isoprene, and 1,3 propanediol showed gradual increases as cancer advanced with 84% accuracy. The genetic mutations, poor blood flow, and rapid growth seen in cancer cells contribute to oxidative stress, and a subsequent increase in reactive oxygen species (ROS) and lipid peroxidation. Lipid peroxidation produces a range of characteristic volatile chemicals such as alkanes and aldehydes like propanal (Barrera, 2012). Another study (n = 173) investigated the reliability of VOCs for colorectal cancer screening and early diagnosis (Altomare et al., 2020). This study found that a combination of age class (above 65 years) and a discrete pattern of 14 VOCs including tetradecane, acetic acid, benzoic acid, and decanal, could discriminate between patients with colorectal cancers from those without cancer with a predictive value of 93%. Three VOCs (ethylbenzene, methylbenzene, and tetradecane) were found to have a highly significant discriminatory ability in detecting patients with colorectal cancer.

**Fig. 6:**
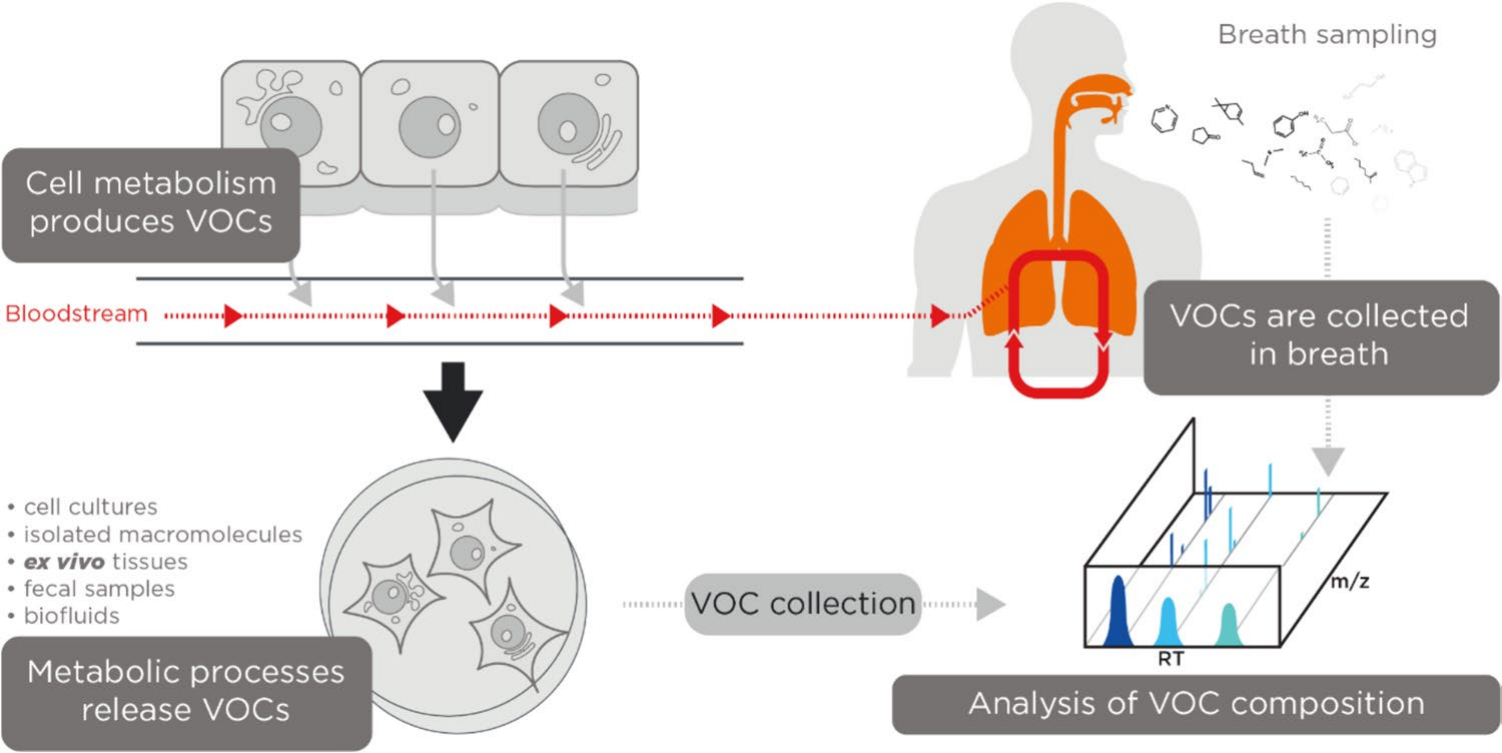
VOCs emitted from in vitro or ex vivo samples can be used as a powerful complementary approach to breath VOC analysis to elucidate the mechanistic origins of VOCs

A recent ex vivo study suggested the VOC pyridine as a potential biomarker of gastric cancer (Mochalski et al., 2023). The study sampled both cancerous tissue and non-cancerous tissues from the same patients and found significantly higher levels of pyridine in the cancerous tissue (Mochalski et al., 2023). Pyridine is a heterocyclic compound that binds nicotinic acid and nicotinamide, which are forms of vitamin B3 (niacin) (Mochalski et al., 2023). Pyridine is a breakdown product that is hypothesized to originate from NADP + , NAD + , and NADPH, compounds that are heavily involved in redox reactions inside cells (Ying, 2008). This suggests that these compounds are being broken down inside cancer cells, producing the unique VOC pyridine that was measured in this study. Validation across diverse gastric tissue to establish robustness and further exploration into breath studies are warranted to ensure it can be reliably measured (Fig. 6).

### Irritable bowel syndrome

Irritable bowel syndrome (IBS) is a chronic condition characterized by altered bowel patterns and abdominal pain. Currently, the diagnosis for IBS is based on the Rome IV Criteria, meaning that IBS cannot be diagnosed if symptoms exist for less than six months, which can result in delayed treatment for IBS patients (Drossman, 2016). A recent study (n = 96) analyzed and compared VOC profiles in both breath and fecal samples of IBS patients split into their different subtypes and healthy controls (Van Malderen et al., 2022). Different subtypes of IBS are described by the dominant stool pattern and include IBS-D (diarrhea), IBS-C (constipation), IBS-M (mixed), and IBS-U (unspecified). This study found that VOCs in breath and feces can differentiate IBS-C patients from the other subtypes and healthy controls, which suggests that there are specific volatiles that could relate to the dominant stool pattern. VOCs in breath and feces were also able to differentiate patients based on symptom severity, quality of life, and visceral sensitivity (Van Malderen et al., 2022), opening the possibility of alternative classifications of IBS patients. The heterogeneity of IBS, with potentially varying underlying pathophysiology, highlights the need to think carefully about study design, and inclusion/exclusion patient criteria in order to confidently identify novel VOC biomarkers. A limitation of this study is that the VOC identities were not revealed, however, the results demonstrated that breath VOCs can differentiate different IBS subtypes, making it a more ideal sampling medium than stool.

### Inflammatory bowel disease

Inflammatory bowel disease (IBD) is another focus for research, particularly in differentiating between IBD and IBS, as both conditions manifest with similar symptoms. There is currently no easy diagnostic test or method to differentiate IBS and the two forms of IBD—Crohn’s disease and ulcerative colitis, with the current gold standard technique for IBD diagnosis involving invasive biopsies from the intestines. A promising compound that could be used as a biomarker specifically to distinguish IBS from IBD was identified as 1-methyl-4-propan-2-ylcyclohexa-1,4-diene, also known as γ-terpinene (Malderen et al., 2020). It is unclear why the exogenous, plant-originating compound increased in IBS subjects but could be the result of gut microbiome composition change, involving a decrease in certain microbes that possess unknown enzymatic activities to metabolize γ-terpinene. Further work is required to determine the specificity and clinical utility of this compound as a biomarker for IBS. Many previous studies have used VOC breath analysis to identify novel biomarkers for IBD (Baranska et al., 2016; Dryahina et al., 2017; Hicks et al., 2015; Patel et al., 2014; Rieder et al., 2016; Smolinska et al., 2018), however, since 2020 there has only been one study (n = 37) utilizing this methodology in IBD to associate the effect of prolonged exercise on inflammation in IBD patients and controls (Henderson et al., 2022). Increased breath butanoic acid levels were found in both patients and controls resulting from prolonged exercise. Butanoic acid is a SCFA with known beneficial impacts on the gut, including anti-inflammatory action (den Besten et al., 2013; Parada Venegas et al., 2019). While there was no specific link to IBD pathophysiology in this study, further investigation into breath SCFAs can potentially inform exercise regimes as therapeutic support in patients with IBD or monitor the progress and predict exacerbations of various inflammatory diseases.

Some IBD patients may develop primary sclerosing cholangitis (PSC), a rare liver disease that commonly remains subclinical until irreversible damage has occurred. A recent study used VOCs in fecal headspace and exhaled breath to detect PSC in an IBD population (n = 73) (van Vorstenbosch et al., 2023b). This study identified phenol, 2-hexanone, styrene, indole, isobutyric acid, nonanal, and 2-propanol as the most important discriminating VOCs for PSC in IBD patients. In the PSC population, some of the metabolites in exhaled breath were found to correlate with the fecal headspace, including ethanol, nonane, 2-octanone, alpha-pinene, benzaldehyde, and limonene. The observed compounds in the fecal headspace relate to microbial dysbiosis, demonstrating that breath analysis can provide a similar window into processes occurring in the gastrointestinal tract (van Vorstenbosch et al., 2023b).

## Liver disease

**Fig. 7:**
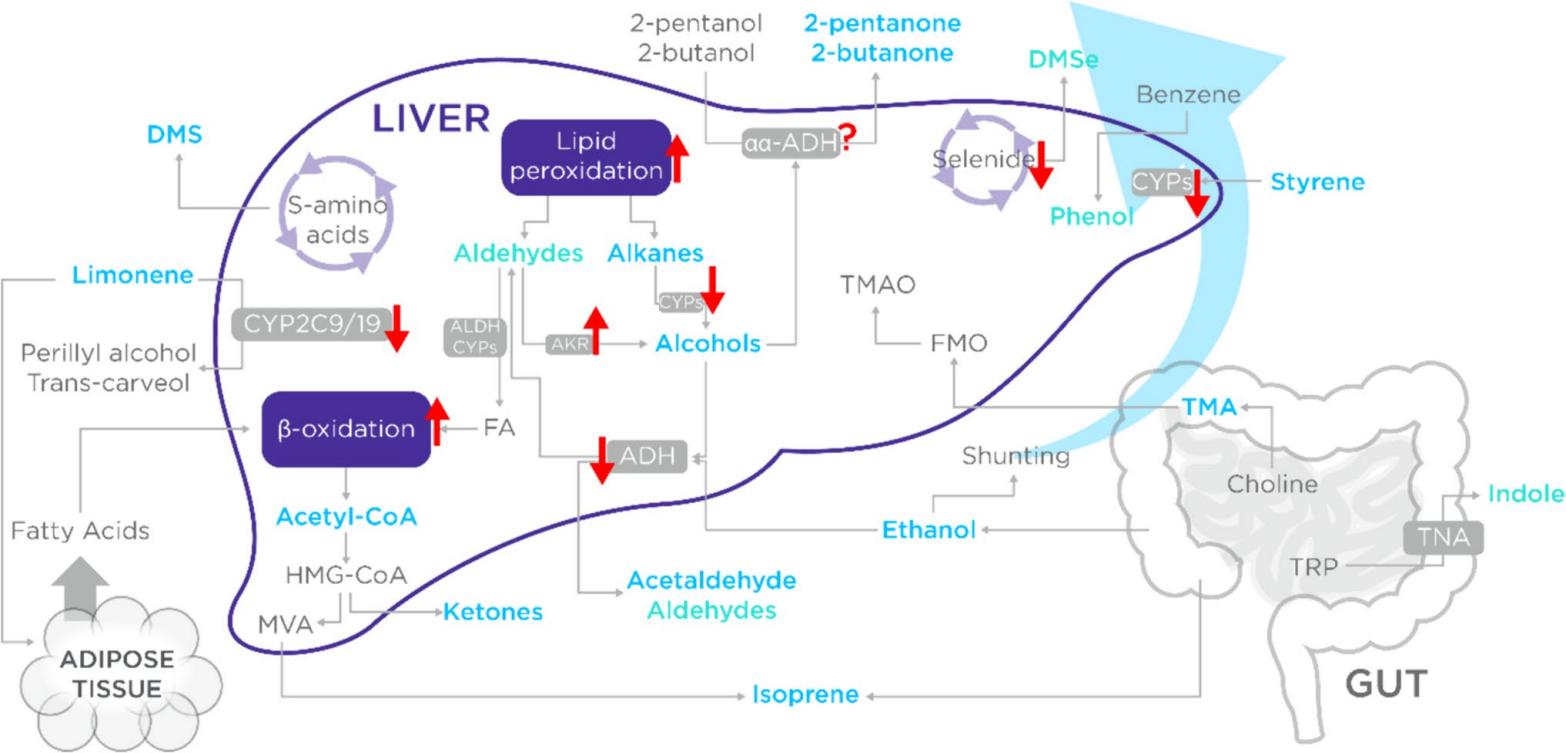
An overview of the metabolic processes ongoing in the liver that can result in VOCs detectable in the breath (labeled in cyan)

Evidence linking breath odor to liver failure dates back over two thousand years to Hippocrates, who described *fetor hepaticus* in individuals with musty breath (Dweik & Amann, 2008). Dimethyl sulfide, the primary compound responsible for *fetor hepaticus,* remained a mystery until the 1970s, when researchers compared breath samples from cirrhotic and healthy subjects using GC (Chen et al., 1970; Kaji et al., 1978; Tangerman et al., 1994). Technological advancements have since enabled numerous studies on chronic liver diseases to identify breath VOC biomarkers (Murgia et al., 2021). The liver’s dense network of blood vessels enhances the feasibility of breath analysis for detecting liver-related VOCs. Despite only being 2.5% of the total body weight, the liver receives 25% of cardiac output, meaning that a large volume of blood is flowing through it at any one time (Lautt, 2009). Blood is supplied via the hepatic artery, which supplies oxygenated blood from the heart, and the hepatic portal vein, which supplies partially oxygenated blood drained from the digestive system. The liver receives blood directly from the digestive system containing nutrients, medication, and toxic substances that must be processed, stored, altered, or detoxified before progressing to the rest of the body (Fig. 7). Because of this high blood supply and high metabolic activity in the liver, VOCs originating from the liver are strongly enriched in the blood, and therefore in the breath through alveolar exchange.

### Metabolic dysfunction-associated fatty liver disease

One recent study (n = 43) targeted 19 VOCs from published literature to distinguish healthy controls from metabolic dysfunction-associated fatty liver disease (MAFLD) patients with or without cirrhosis (Sinha et al., 2020). Combining VOCs dimethyl sulfide and limonene enabled discrimination of cirrhotic-MAFLD patients from healthy controls (AUC = 0.98), as well as cirrhotic MAFLD from non-cirrhotic MAFLD patients (AUC = 0.91). While a small pilot study, it suggested the potential of VOCs for earlier liver disease diagnosis. However, most breath VOC research to date has focused on later-stage diseases like cirrhosis or liver cancer. This is likely due to fewer non-targeted biomarker discovery studies being conducted in earlier disease stages, as recruitment may be slow in diseases with little or no symptoms. It is also possible that the disease-relevant compounds are of low abundance and therefore below the detection limit. Finally, the lack of standardization in breath collection and analysis, including the differentiation of breath and background compounds, may have impeded the identification of novel biomarkers in earlier stages.

### Liver cirrhosis

One study (n = 88) conducted an untargeted VOC analysis from breath samples of cirrhotic patients and controls (Ferrandino et al., 2023a) and found that limonene, along with 28 other breath VOCs, significantly differed between cirrhosis patients and controls. Increased levels of limonene have been found in liver disease patients in several studies (Fernández del Río et al., 2015; Ferrandino et al., 2023a; Ferrandino et al., 2020; Patnaik et al., 2022). Limonene is a VOC with exogenous origins commonly found in certain fruits and citrus products, metabolized by the cytochrome P450 (CYP) family, particularly CYP2C9 and CYP2C19 in the liver. Therefore, the findings imply that impaired liver metabolism increases the amount of the compound excreted in the lungs.

The repeated findings of increased breath limonene in cirrhosis led to a longitudinal study (n = 60) administering 100 mg limonene substrate to overnight-fasted cirrhotic patients and controls (Ferrandino et al., 2023b). Upon observing peak levels of limonene in both groups after 20 min post-administration, limonene levels returned to baseline after two hours while showing a slower decline in cirrhotic patients. The best-performing timepoint was observed at 60 min post-administration (AUC = 0.91). Limonene was found to correlate with Model for End-Stage Liver Disease (MELD) scores and fibrosis indicators in the cirrhosis group, and interestingly, it was associated with portal hypertension. This study showed for the first time the application of an EVOC as a probe to facilitate non-invasive cirrhosis detection. This also implies that administering safe EVOCs to patients is an innovative approach to breath analysis. With dietary intake (or fasting hours) standardized prior to testing, EVOCs can be implemented in primary care to enhance disease diagnosis.

### Liver cancer

The potential of VOC biomarkers for diagnosis in other liver diseases, such as hepatocellular carcinoma (HCC), has also been demonstrated (Acevedo-Moreno et al., 2018; Miller-Atkins et al., 2020; Qin et al., 2010; Sukaram et al., 2022). This also includes the aforementioned VOC limonene, which showed a correlation with serum bilirubin and albumin levels in subjects with cirrhosis-induced HCC in one recent study (n = 84) (Ferrandino et al., 2020). However, when building predictive models with breath VOCs to classify liver diseases, the inclusion of a broader disease category as part of the analysis can help understand the model limitations and disease misclassification better. This mirrors real-world VOC breath test applications, which will likely screen populations with various underlying diseases, including different liver diseases exhibiting similar symptoms. One study investigated a total of 296 patients with various diseases, including cirrhosis, HCC, colorectal cancer liver metastases (CRLM), pulmonary hypertension (which sometimes results in congestive hepatopathy), and controls with no liver diseases (Miller-Atkins et al., 2020). Twenty-two VOCs commonly found in human breath that have been associated with different diseases were selected for analysis, resulting in a predictive model that classified patients with 85% accuracy. Acetaldehyde and acetone, which increased in cirrhosis and HCC patients, were considered important VOCs for differentiating disease and control groups in the predictive model. More importantly, the study showed that the predictive model has better sensitivity to detect HCC than alpha-fetoprotein (AFP), a biomarker currently widely used but only secreted in roughly 50% of HCC tumors (Forner et al., 2018).

Another study also compared the diagnostic performance of HCC between breath VOCs and AFP with similar findings (Sukaram et al., 2022). The study (n = 343) identified five VOCs (ethanol, acetone dimer, benzene, 1,4-pentadiene, and isopropyl alcohol) that can distinguish early and advanced HCC patients with 82.6% accuracy. Of these five compounds, acetone dimer presented higher AUCs than AFP regardless of the disease stage being compared to, suggesting better overall diagnostic performance. The study also found significantly lower acetone dimer levels in HCC patients who responded to cancer therapy, suggesting breath VOCs are feasible for treatment monitoring. Because acetone can be reduced to isopropyl alcohol by alcohol dehydrogenase, the two VOCs are directly correlated and usually co-exist. This also means that isopropyl alcohol, while commonly known as a compound from cleaning products, can also be produced endogenously (Janfaza et al., 2019; Li et al., 2017; Wang et al., 2021). Interestingly, the study found that isopropyl alcohol may be used as a prognostic biomarker, as increased levels of isopropyl alcohol were significantly associated with declined survival of HCC patients. It should be noted that most of the HCC patients in the study had chronic viral hepatitis B infection; therefore, it remains unknown if the VOCs identified apply to HCC from hepatitis C or alcoholic cirrhosis due to possible different dysregulated pathways. Additionally, given the breath sampling and analytical method used in the study, exogenous VOCs from the environment may potentially be a confounding factor. Regardless, the study demonstrated the potential of breath VOCs for HCC diagnosis and treatment monitoring.

While standardizing breath analysis is crucial, several factors are pivotal in identifying VOC biomarkers for liver diseases. In liver cancer, focusing on cancer-specific VOC profiles is essential to differentiate from other cancers (Janfaza et al., 2019). For chronic liver diseases, due to it often being asymptomatic, it is necessary to identify VOCs from early-stage liver diseases such as metabolic-associated steatohepatitis (MASH) or MAFLD. Finally, while challenging, conducting longitudinal studies in chronic liver diseases can help understand VOC concentration changes throughout disease progression (Younossi et al., 2018).

## Drug development

Breath VOC biomarkers have values beyond clinical practice, especially in enhancing clinical research through providing valuable insights throughout drug development stages, including preclinical phases, clinical trials, personalized medicine, and regulatory approval. However, selecting suitable breath biomarkers remains challenging due to limited research regarding VOC metabolism and its linkage to cellular processes.

### Preclinical phases

Most cell culture studies have focused on the presence of compounds (Filipiak et al., 2016; Issitt et al., 2022b; Jia et al., 2019). This potentially overlooks VOCs utilized by cells as substrates, especially in nontargeted analysis where the detection limit (ppbv) is higher than in targeted approaches (pptv). Understanding the systemic uptake and release of VOCs in normal and disease cell processes is crucial for developing clinical biomarkers. One recent study took a novel approach in headspace analysis to understand VOC uptake/release by quantifying the rate of VOC metabolism through multiple time points (Issitt et al., 2022a). The authors demonstrated that within the VOCs selected for this study, the quantified fluxes (both uptake and release) in metabolism differ among different cell types and statuses (disease). More importantly, the treatment of Doxorubicin, a chemotherapeutic agent, resulted in significant alterations in VOCs metabolized by the cells. Because the dynamics of VOCs are reflective of cellular metabolisms, the link to phenotype and pathophysiology not only provides potential targets for diagnostic research but also an important avenue of research in drug development.

In in vivo studies, analyzing breath VOCs from mice offers several benefits such as controlled environments to reduce ambient air variation, and inbred mice strains to minimize inter-individual variation. However, collecting enough breath for analysis in murine models with minimal stress induction has been challenging. A recent publication presented a novel device for non-invasive breath collection in mice, bypassing the need for anesthetics (Hintzen et al., 2022). Future development of robust methods that enhance control over signal-to-noise ratios will enable the detection of VOCs with low abundance in mice, increasing the opportunities for novel biomarker discovery. Comparative studies in humans and mice can help translate findings to clinical settings.

### Clinical trials

A study (n = 89) has demonstrated how breath VOCs can be utilized to monitor treatment response (Bannier et al., 2022). The study was conducted on asthmatic preschool children with inhaled corticosteroid (ICS) treatment, and the results showed significantly different VOC profiles from responsive and unresponsive children (Bannier et al., 2022). Although the sample size was too small for a prediction model, the study showed that ICS treatment significantly alters exhaled VOCs. It also indicated that pharmacological treatment, including dosage and timing of medication, should be considered in breath studies. The ability of breath analysis to better identify wheezing children who might benefit from ICS can potentially prevent both over and undertreatment with ICS.

Breath VOCs can also be useful for monitoring drug pharmacokinetics/pharmacodynamics. One study (n = 78) conducted breath and urine analysis in asthma patients with drug treatment to understand the relationship between breath VOCs and drug usage (Brinkman et al., 2020). The findings demonstrated an association between excreted traces of asthma medication salbutamol and oral corticosteroids (OCSs) in urine and exhaled breath VOCs. Despite potential data variation due to the study’s multi-center design, the connection was validated in an independent cohort. This suggests that breath VOCs have great potential for monitoring drug pharmacokinetics/pharmacodynamics, and well-documented standardized operating protocols and breath collection techniques across different sites are crucial for revealing the potential applications of breath VOCs.

To accelerate the application of VOC biomarkers in drug development, understanding the link between cellular processes and VOC metabolisms is important for identifying biomarkers. These biomarkers must also be translatable to preclinical and clinical settings. Standardizing breath analysis, including the confirmation of compound identity through chemical standards, will facilitate biological interpretation and help generate new research hypotheses.

## Conclusions

In this review, we have discussed key methodological considerations for breath researchers to consider for better standardization, and hence better translational opportunities for clinical tests, as well as some highlights of progress that have advanced the field since 2020. Many of the disease-associated VOCs are suggested to originate from lipid peroxidation, supporting previous conclusions that it is a key producer of VOCs. It is yet to be seen whether lipid peroxidation signatures between different diseases are significant enough to provide robust differential diagnosis. A greater understanding of breath complexity and the identification of specific VOCs that are hallmarks of specific processes could lead to the level of specificity required for the development of clinically useful tests. If the association between the production of VOCs in the body, their presence in breath, and the relationships between specific VOCs and physiological states are established, this could support breath as a robust metabolomic platform for clinical biomarker analysis, similar to blood, urine, and fecal sampling. As breath can be collected non-invasively and is inexhaustible, this would support the development of next-generation diagnostic, and biomarker monitoring technology to address major remaining clinical needs, and ultimately save lives.

## Data Availability

No datasets were generated or analysed during the current study.
